# Mixed Acid Fermentation of Carbohydrate-Rich Dairy Manure Hydrolysate

**DOI:** 10.3389/fbioe.2021.724304

**Published:** 2021-08-03

**Authors:** Abel T. Ingle, Nathaniel W. Fortney, Kevin A. Walters, Timothy J. Donohue, Daniel R. Noguera

**Affiliations:** ^1^ Department of Civil and Environmental Engineering, University of Wisconsin-Madison, Madison, WI, United States; ^2^ Wisconsin Energy Institute, University of Wisconsin-Madison, Madison, WI, United States; ^3^ Great Lakes Bioenergy Research Center, Madison, WI, United States; ^4^ Department of Bacteriology, University of Wisconsin-Madison, Madison, WI, United States

**Keywords:** dairy manure, biomass pretreatment, medium-chain fatty acids, mixed culture fermentation, lignocellulosic biomass

## Abstract

Dairy manure (DM) is an abundant agricultural residue that is largely composed of lignocellulosic biomass. The aim of this study was to investigate if carbon derived from DM fibers can be recovered as medium-chain fatty acids (MCFAs), which are mixed culture fermentation products of economic interest. DM fibers were subjected to combinations of physical, enzymatic, chemical, and thermochemical pretreatments to evaluate the possibility of producing carbohydrate-rich hydrolysates suitable for microbial fermentation by mixed cultures. Among the pretreatments tested, decrystalization dilute acid pretreatment (DCDA) produced the highest concentrations of glucose and xylose, and was selected for further experiments. Bioreactors fed DCDA hydrolysate were operated. Acetic acid and butyric acid comprised the majority of end products during operation of the bioreactors. MCFAs were transiently produced at a maximum concentration of 0.17 mg COD_MCFAs_/mg COD_Total_. Analyses of the microbial communities in the bioreactors suggest that lactic acid bacteria, *Megasphaera*, and *Caproiciproducens* were involved in MCFA and C4 production during DCDA hydrolysate metabolism.

## Introduction

Raw dairy manure (DM) is an abundant organic waste stream produced at rates of hundreds of megatonnes per year (Fischer, 1998; Milbrandt, 2005; Nass, 2007). A common route for DM management is storage (e.g., slurry tanks or lagoons) followed by land application. This is a convenient approach, but contributes to greenhouse gas (GHG) emissions (Aguirre-Villegas and Larson, 2017). Anaerobic digestion (AD) can measurably reduce GHG emissions from DM while offsetting uncontrolled biodegradation that would otherwise take place during storage or other management processes (Aguirre-Villegas and Larson, 2017). AD is an established bioprocess technology to convert organic material in DM into biogas (i.e., methane, carbon dioxide, and other trace gases). Captured biogas can be combusted for heat and electricity or, if upgraded, used for injection in the natural gas grid or used as transportation fuel. In the US, such conversions (i.e., into electricity or natural gas) are considered renewable fuel per the incentivizing Renewable Fuel Standard program created under the Energy Policy Act of 2005 (United States of America, Senate and House of Representatives, 2005); however, their long-term economic viability is currently a suspected bottleneck in the wide-spread adoption of anaerobic digestion (Zaks et al., 2011; Von Keyserlingk et al., 2013). Thus, the conversion of manure into products of higher economic value and broader applications is of current research interest. To date, biogas is the only bioproduct derived from DM that is produced at an industrial scale, though some ethanol plants plan to incorporate DM-fed AD to achieve a closed-loop system (Devuyst et al., 2011). The liquid fraction of DM consists largely of volatile fatty acids produced, but not absorbed, in the cow gut, making it a suitable substrate for AD (Rico et al., 2007). The solid (or lignocellulosic) fraction of DM is mainly composed of recalcitrant plant biomass fibers, existing still in a lignocellulosic matrix that resisted degradation in the cow’s digestive system, making it inherently of low biodegradability.

We are interested in determining whether the lignocellulosic fibers in the solid fraction of DM can be used as a feedstock for conversion of DM to other fermentation products of potential economic interest, such as medium-chain fatty acids (MCFAs), which are five-to eight-carbon monocarboxylic acids that can supplement livestock feed (Mills et al., 2010), be used as antimicrobials (Kim and Rhee, 2013), or be chemically upgraded as renewable fuel additives (Agler et al., 2011; Urban et al., 2017). It has been shown that anaerobic self-assembled microbiomes can produce MCFAs from diverse organic feedstocks (Stamatopoulou et al., 2020). Successful MCFA production within these microbiomes relies on the proper enrichment of bacteria capable of chain elongation, a metabolic process by which certain intracellular fermentation intermediates (e.g., acetyl- and propionyl-CoA) are extended by two carbons *via* a cyclic pathway known as reverse beta-oxidation (Han et al., 2018). In this research, we hypothesized that the chemical energy within the lignocellulosic fraction of DM can be recovered as MCFAs. We explored a variety of pretreatment strategies with the objective of producing a DM hydrolysate that was rich in easily fermentable carbohydrates. Mixed culture fermentations were performed using DM hydrolysate from the pretreatment that produced the highest concentration of glucose and xylose, and the resulting microbial communities were analyzed to determine the key community members that participate in the conversion of the lignocellulosic fraction of DM into MCFAs and other fermentation products.

## Materials and Methods

### Dairy Manure Collection and Bioprocessing

**FIGURE 1:**
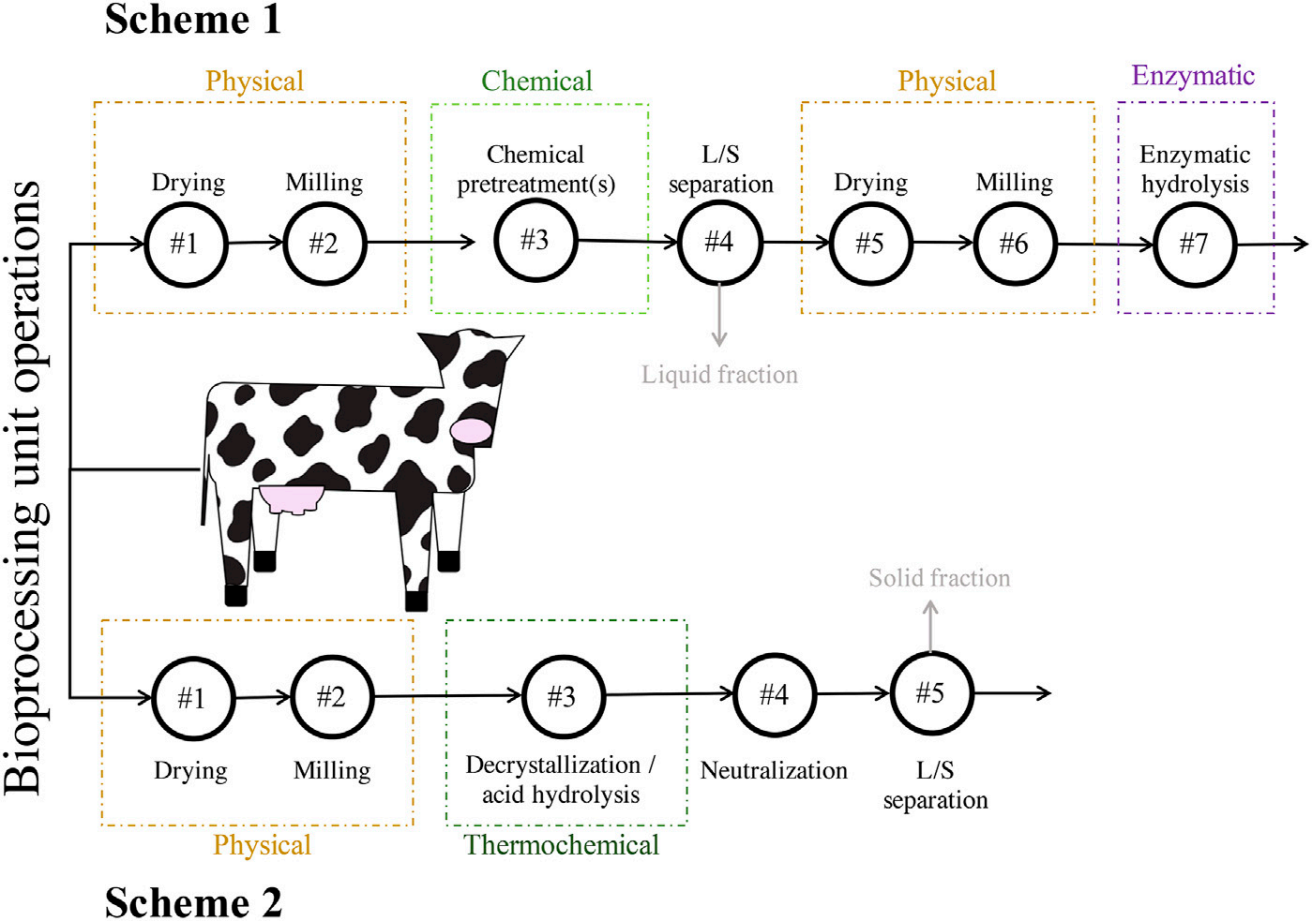
Strategies for pretreatment of the lignocellulosic fraction of DM used in this study. Scheme 1 consisted of seven total unit operations and included physical, chemical, and enzymatic steps. Scheme 2 consisted of five total unit operations and included physical and thermochemical steps. The grey arrows and text indicate streams of material that were neither used in downstream bioprocessing units nor analyzed. Abbreviations: liquids-solids (L/S).

Batches of DM were obtained from a single dairy cow, immediately after excretion, at the Dairy Cattle Center housed at the University of Wisconsin-Madison. The manure was stored at 4°C until physical pretreatment. Two different schemes were used for the pretreatment of the lignocellulosic fraction of DM, a part of the solid fraction hereafter referred to as DM fibers. The first scheme (Scheme 1) involved physical, chemical, and enzymatic steps. The second scheme (Scheme 2) involved physical and thermochemical steps and did not require enzymatic hydrolysis (Figure 1). The two schemes used separate batches of DM that were collected several months apart.

### Drying, Milling, and Chemical Pretreatments

Collected DM was dried at 101°C for 24 h. Dried DM was then milled to pass through a 1-mesh screen using a laboratory hammer mill (Model No. 1024XC, Christy & Norris Ltd., England). Five different chemical pretreatments were tested in 400-ml shaker-flasks using dried, milled DM at a solids loading of 10% (100 g/L) in working volumes of 150 ml of chemical liquor. The pretreatments were: 1) sulfite pretreatment to overcome recalcitrant lignocellulose (SPORL); 2) dilute acid (DA); 3) alkaline; 4) copper-alkaline-hydrogen peroxide (Cu-AHP); and 5) copper-alkaline-hydrogen peroxide with alkaline pre-extraction and batch addition of hydrogen peroxide (CAP); where DA and SPORL followed Yang et al. (2015) methods and alkaline, Cu-AHP, and CAP followed Bhalla et al. (2016) methods. Chemical loadings, yields, and durations of chemical pretreatment are reported in Table 1. After chemical pretreatments, solids were separated from the spent liquor, twice washed (150 ml of deionized water), separated from wash volumes, and dried at 100°C for 2 h, remilled, then stored at 4°C until subject to enzymatic hydrolysis, and are referred to hereafter as pretreated DM fibers of Scheme 1 (Figure 1). The 2,2′-bipyridine and copper sulfate, pentahydrate used during pretreatment (Table 1) were purchased from Alfa Aesar (Haverville, MA). Sulfuric acid (98%), hydrogen peroxide (30%), sodium hydroxide, and sodium sulfite were purchased from FisherSci (Pittsburgh, PA). Solid-liquid separation was carried out using centrifugation (10 min at 10,000 relative centrifugal force). One-way analysis of variance (ANOVA) with Tukey posthoc testing was used to determine the statistical significance (alpha = 0.05) of cellulose, hemicellulose, and lignin fractions between untreated and pretreated DM fiber samples (Supplementary Table S1), and was performed using GraphPad Prism software (v8.4.2).

**TABLE 1:** Summary of chemical pretreatments in Scheme 1 and their effect on the lignocellulosic composition of resultant fibers.

Pretreatment method	Chemical loading (% wt_chemical_/wt_driedDM_)^a^						Duration (hrs)	Solids retained (%)	Average composition of pretreated DM fibers (g/100g DM fiber treated)			
	SA	SS	SH	Cu^2+^	2bp	HP			Cellulose	Hemi-cellulose	Lignin	N^c^
Untreated^b^	0	0	0	0	0	0	0	100	21.1	14.1	9.5	4
DA	4	0	0	0	0	0	1	80.0	20.7	13.9	14.6	2
SPORL	4	9	0	0	0	0	1	81.3	22.0	14.1	13.1	2
Alkali	0	0	10	0	0	0	24	80.7	19.9	8.8^d^	10.2	2
Cu-AHP	0	0	10	0.13	0.62	10	24	81.3	21.6	8.6^d^	11.3	2
CAP	0	0	10	0.06	0.31	10	1, 23	54.7	19.0	4.5^d^	6.1	2

a Abbreviations: Sulfuric acid (SA), sodium sulfite (SS), sodium hydroxide (SH), copper (Cu^2+^), 2,2′-bipyridine (2bp), hydrogen peroxide (HP).

b Untreated refers to dried, milled samples that were not subjected to any pretreatment and were taken from the same batch of DM as pretreated DM.

c N indicates the number of biological replicates involved.

d Values that possess a statistical difference when compared to untreated sample values (Supplementary Table S1).

### Enzymatic Hydrolysis

In a Certomat® BS-1 incubation shaking cabinet (Sartorius AG 85030-520-51, Göttingen, Germany), enzymatic hydrolysis tests of dried, milled, chemically pretreated, re-dried, re-milled DM were carried out in 500-ml Erlenmeyer flasks at working volumes of 100 ml of buffer (50 mM sodium acetic acid, 30 mM sodium azide, pH 5.30) and 10% solids loading. A temperature of 50°C and shaking rate of 180 revolutions per minute (RPM) were maintained during the experiments. An enzyme loading of 50 filter-paper-units/g cellulose was applied using commercially available Cellic® CTec 2, an enzyme complex cocktail primarily known for cellulase that also demonstrates xylanase, cellobiohydrolase, and β-glucosidase activities (Sun F. F. et al., 2015), by Novozymes (Bagsvaerd, Denmark), purchased through Sigma Aldrich (St. Louis, MO). Incubations lasted 72 h. Glucose and xylose concentrations during incubations are reported in Supplementary Table S2.

### Thermochemical Pretreatment

In a second pretreatment scheme (Figure 1), a two-phase thermochemical pretreatment, first described in Liao et al. (2006), was applied to DM fibers instead of chemical and enzymatic pretreatment. In a first phase, DM fibers were chemically loaded with 75% sulfuric acid on a 5-to-3 (acid solution-to-DM fibers) weight basis. For 30 min, the mixture was manually mixed using glass mortar and pestle. In the second phase, the mixture was placed into a 4,000-ml boiling flask and deionized water was added until an acid concentration of 12.5% was reached. The boiler flask was heated until the mixture reached 103°C, which required 15–20 min of ramp up time depending on the size of the batch. To neutralize the pretreatment mixture [hereafter, referred to as decrystallized-dilute acid (DCDA) hydrolysate] 5 M NaOH was slowly added in a fumehood until a pH of ∼5.50 was reached. After hours of settling, the liquid fraction of the mixture was retained for downstream processes. Details of the chemical loadings and other aspects of this pretreatment are reported in Supplementary Table S3.

### Bioreactor Operations

Fermentation studies were carried out at mesophilic (35°C) conditions either in batch or flow-through continuously stirred tank reactors. All bioreactors were seeded with sludge from an acid-phase digester at the Nine Springs Wastewater Treatment Plant (Madison, Wisconsin). Between inoculations, the sludge inoculum was stored at 4°C. A batch bioreactor was ran for 14 days at a working volume of 48 ml and was sealed with a rubber stopper. A long-term flow-through reactor was ran for 42 days. A short-term flow-through reactor was ran for 10 days. The flow-through bioreactors (consisting of a 400-ml glass vessel with pumps to control feed and withdrawal of liquid) with a working volume of ∼300 ml were mixed at 150 RPM with a magnetic stir bar, and operated with a residence time of 6 days by pumping out 1.9 ml of fermentation broth and replacing it with hydrolysate every hour. The bioreactors were sealed with a rubber stopper that included ports for sampling (valved), influent flow, effluent flow, and pH control. A pH of 5.50 was maintained by automated addition of 5 M sodium hydroxide (NaOH). All bioreactors were operated at different times.

### Chemical Analyses

DM fiber samples from Scheme 1 were submitted to the University of Wisconsin Soil and Forage Laboratory (Marshfield, Wisconsin) for measurements of neutral detergent fiber (NDF, which comprise hemicellulose, cellulose, and lignin), acid detergent fiber (ADF, which comprise cellulose and lignin), lignin, total starch, and soluble crude protein (sCP) content (UW Soil and Forage Lab, 2013) where NDF, ADF, and lignin were measured in both untreated and pretreated samples and total starch and sCP were only measured in untreated. Hemicellulose was determined by subtracting values of ADF from NDF. Cellulose was determined by substracting values of lignin from ADF. Measurements of chemical oxygen demand (COD) and soluble COD (sCOD) were performed using High-Range COD Digestion Vials (Hach 2125915, Loveland, CO, United States) as per standard methods (American Water Works Association and Water Environment Federation, 2005). Procedures to quantify total suspended solids (TSS) and volatile suspended solids (VSS) in DCDA hydrolysates were performed according to Method 2540D and Method 2540G, respectively (American Water Works Association and Water Environment Federation, 2005). Total soluble carbohydrates in DCDA hydrolysates and bioreactor samples were measured with the anthrone method (Yemm and Willis, 1954). Total soluble proteins in DCDA hydrolysates were measured (Smith et al., 1985) with the bicinichoninic acid assay using the Pierce BCA Assay Kit and the Compat-Able Protein Assay Preparation Reagent Set. Ammonia nitrogen in DCDA hydrolysates was quantified spectrophometrically (wavelength = 655 nm) after properly mixing samples with Ammonia Salicylate (Hach 2395266, Loveland, CO, United States) and Ammonia Cyanurate (Hach 2395466, Loveland, CO, United States) Perchmachem® pillow reagent reagent powders. Phosphate phosphorous in DCDA hydrolysates was quantified spectrophometrically (wavelength = 890 nm) after properly mixing sample with PhosVer® 3 Phosphate reagent (Hach 2106069, Loveland, CO, United States).

Samples from all DCDA hydrolysates and bioreactors were centrifuged for 10 min at 10,000 relative centrifugal force, then filtered using 0.22-μm syringe filters (ThermoFisher Scientific SLGP033RS, Waltham, MA, United States) and used for quantification of lactic, acetic, propionic, butyric, valeric, hexanoic, heptanoic, and octanoic acids; D-glucose, xylose, ethanol, cellobiose, and sulfate and sodium ions. Samples used to quantify sCOD and soluble carbohydrates were obtained in the same way.

Glucose, xylose, cellobiose, and acetic acid in DCDA hydrolysate and DL-lactic acid in fermentation broths of the two flow-through bioreactors were analyzed with high-performance liquid chromatography (HPLC) and quantified with an Agilent 1260 Infinity refractive index detector (Agilent Technologies, Inc. Palo Alto, CA) using a 300 × 7.8 mm Bio-Rad Aminex HPX-87H column and a Cation-H guard column (BioRad, Inc., Hercules, CA). A column temperature of 50°C was used and 0.02 N H_2_SO_4_ was used for the mobile phase with a flow rate of 0.50 ml min^−1^. A YSI 2700 Series Biochemistry Analyzer (YSI Inc., Yellow Springs, OH) was used to quantify L-lactic acid, ethanol, D-glucose, and xylose in aliquots of fermentation broths from all bioreactors using immobilized membranes of L-lactic acid oxidase, alcohol oxidase, glucose oxidase, and pyranose oxidase, respectively.

The concentrations of monocarboxylic acids (acetic, propionic, butyric, valeric, hexanoic, heptanoic, and octanoic) in fermentation broths were determined using gas chromatography with a flame ionization detector (GC-FID) using a Shimazdu 2010 GC System (Shimazdu Co., Kyoto, Japan) equipped with an AOC-20i autosampler. The GC column was a high-performance capillary column with dimensions of 30 m × 0.53 mm × 1.00 μm (Zebron ZB-FFAP, Phenomenex, Torrance, CA) for underivatized acids and alcohols (Vasquez et al., 2014; Chwialkowska et al., 2019). Samples were diluted with a dilute phosphoric acid solution prior to sampling.

Sulfate and sodium concentrations were quantified *via* ion chromatography (ICS-2100 and ICS-1100, Dionex, Sunnyvale, CA, United States). For sulfate, an IonPac AS11-HC 250 mm × 4 mm analytical column and an IonPac AG11-HC 50 mm × 4 mm guard column (both from Dionex, Sunnyvale, CA, United States) were used in series with a 30 mM NaOH mobile phase at a flow rate of 4 ml min^−1^, and column temperature of 30°C. For sodium, an IonPac CS12A 250 mm × 4 mm analytical column and an IonPac CG21A 50 mm × 4 mm guard column (both from Dionex, Sunnyvale, CA, United States) were used in series with a 20 mN methanesulfonic acid mobile phase at a flow rate of 4 ml min^−1^, and column temperature of 30°C.

### Bacterial Community Analysis

DNA was extracted from the seed sludge and bioreactor samples using a PowerSoil® Pro DNA Isolation Kit (MoBIO Laboratories 12,888, Carlsbad, CA). Extracted DNA was quantified using a Qubit 3.0 (Thermo Fisher Scientific Q33126, Waltham, MA). The V3 and V4 hypervariable regions of the 16S rRNA gene were amplified by University of Wisconsin-Madison Biotechnology Center (UWBC; https://www.biotech.wisc.edu/) with fusion set primers [forward primer 341f: 5′-ACACTCTTTCCCTACACGACGCTCTTCCGATCT(N)_0/6_CCTACGGGNGGCWGCAG-3′, reverse primer 805r: 5′-GTGACTGGAGTTCAGACGTGTGCTCTTCCGATCT(N)_0/6_GACTACHVGGGTATCTAATCC-3′]. Amplicons were sequenced on an Illumina MiSeq sequencer (Illumina, San Diego, CA) using pair-end 300 base pair kits at UWBC. Microbial analyses of sequencing data were processed with the Quantitative Insights Into Microbial Ecology 2 (QIIME 2) pipeline (Bolyen et al., 2019). Low-quality reads and inferred chimeras were removed from raw sequences with the denoising DADA2 pipeline (Callahan et al., 2016) using the following input parameters to identify amplicon sequence variants (ASVs), which are differentiated from operational taxonomic units (Callahan et al., 2017): p-trim-left-f 18, p-trim-left-r 22, p-trunc-leng-f 301, p-trunc-len-r 281. The SILVA database Release 132 (https://www.arb-silva.de/) was used for reference taxonomy (Quast et al., 2012). Taxonomic assignment of ASVs was carried out using a naïve-Bayes classifier trainer in which only classifications with confidence values greater than 0.7 were retained, as recommended (Bokulich et al., 2018). For phylogenetic tree construction, rarified denoised sequences were aligned with MUSCLE (v3.8.31) (Edgar, 2004), then maximum-likelihood phylogeny was built with RAxML (v8.2.11) using the GTRGAMMA method with 1000 bootstraps (Stamatakis, 2014). Interactive Tree of Life (ITOL) v5 was used for tree visualization (Letunic and Bork, 2021). The *superheat* R package was used for heatmap visualizations (Barter and Yu, 2018). Generalized least square models were generated to statistically analyze microbial data (ASVs collapsed at the genus level) and metabolomic data of each bioreactor using the *gls* function in the *nlme* package in R (Pinheiro et al., 2017). In these models, time was correlated to all predictor variables using the corAR1 structure. Redundancy analysis was performed as described in Scarborough et al. using relative frequency (or relative abundance) of individual ASVs from the two flow-through bioreactors as the species matrix (Scarborough et al., 2018).

## Results

### Pretreatment of DM Fibers

**FIGURE 2:**
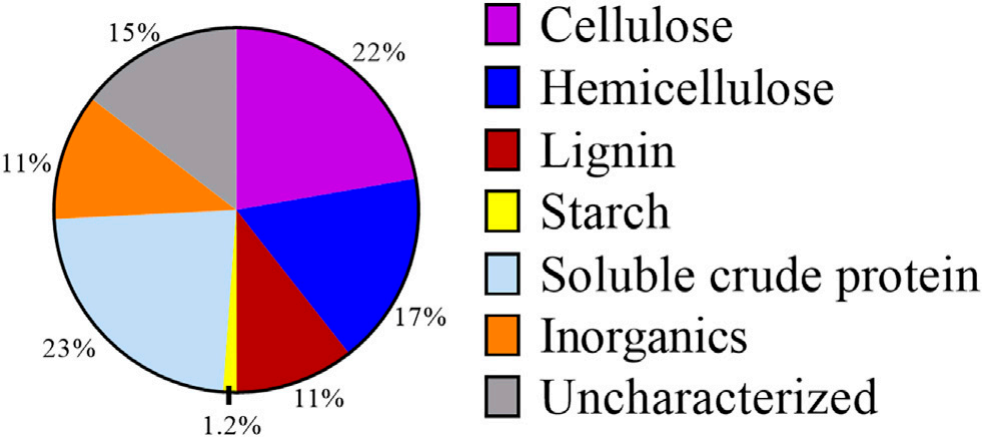
Composition of DM fibers on a weight basis. Percentages shown are averages from both Scheme 1 and Scheme 2 batches of DM.

As DM fibers exhibit lower biological degradation than other complex organic substrates (Labatut et al., 2011), we explored conventional and unique bioprocessing strategies to evaluate if it could be used as a substrate for microbial fermentations. A composition analysis of DM fibers (Figure 2) indicated the presence of cellulose (22% w/w of DM), hemicellulose (17%), and starch (1.2%) as the main carbohydrate components. Other major components of DM fibers included sCP (23%) and lignin (11%). This composition is typical of DM (Rico et al., 2007), and the high fraction of carbohydrates in the DM fibers suggests the possibility of pretreatment combinations to release hexoses and pentoses that could be used in microbial fermentations.

DM fibers were subjected to different unit operations intended to either increase their enzymatic hydrolysability while retaining most of the fibers as solids (Scheme 1, Figure 1) or directly breakdown the complex carbohydrates into its soluble monomeric units without the need of enzymatic hydrolysis (Scheme 2, Figure 1). All chemical pretreatment processes, except for CAP, retained approximately 80% of the original weight in the solid fraction before enzymatic hydrolysis (Table 1), whereas the CAP process solubilized a large fraction of the DM fibers, with only 55% of solids remaining after pretreatment. The compositions of resulting DM fibers after each chemical pretreatment were measured (Table 1) and their differences were assessed (Supplementary Table S1). The resultant material of each Scheme 1 process had a higher fraction of cellulose than untreated DM (i.e., cellulose-enriched), showing no significant loss of cellulose during chemical pretreatment. Whereas the dilute acid-based pretreatments (DA, SPORL) resulted in hemicellulose-enrichment and no apparent solubilization of hemicellulose. Alkaline-based pretreatments (Alkaline, Cu-AHP, CAP) showed significant differences when compared to untreated DM fibers (Table 1). The absolute cellulosic mass of all chemically pretreated DM fibers remained comparable to untreated DM at ∼ 20% (g cellulose/g treated DM fiber) in all chemical pretreatments. CAP produced the most cellulose-enriched DM fibers, and also led to the most significant loss in hemicellulose (Table 1), making it undesirable within the Scheme 1 pretreatments in which the last unit operation is enzymatic hydrolysis of the retained solids for the recovery of xylose in addition to glucose (Figure 1).

The concentrations of glucose and xylose in filtered hydrolysates made by chemical-enzymatic pretreatment (after unit operation #7 in Scheme 1, Figure 1) and thermochemical-based pretreatment (after unit operation #5 in Scheme 2, Figure 1) were measured (Table 2). Dilute acid-based pretreatments resulted in higher concentrations of glucose and xylose than the three alkali-based pretreatments tested (Alkaline, Cu-AHP, CAP). Furthermore, during enzymatic hydrolysis, glucose and xylose concentrations decreased in the alkaline pretreated liquors (Supplementary Table S2), an observation that suggests formation of reaction products from residual chemical pretreatment liquor as it is known such chemistry may occur (Forssk et al., 1976).

**TABLE 2:** Glucose and xylose concentrations in DM hydrolysates produced using various pretreatments.

Scheme	Pretreatment	Glucose^a^ (g/L)	Xylose^a^ (g/L)	CGC^a^ ^,^ ^b^ % (w/w)	HXC^a^ ^,^ ^b^ % (w/w)
Scheme 1	Untreated	2.90 ± 0.01	3.11 ± 0.02	17.4	27.7
Scheme 1	DA	5.3 ± 0.2	4.90 ± 0.02	25.8	35.1
Scheme 1	SPORL	6.1 ± 0.1	6.40 ± 0.18	28.0	45.9
Scheme 1	Alkaline	0.65 ± 0.01	0.13 ± 0.02	3.30	1.47
Scheme 1	Cu-AHP	0.00 ± 0.00	0.12 ± 0.01	0.00	1.44
Scheme 1	CAP	0.17 ± 0.01	0.28 ± 0.01	0.62	4.21
Scheme 2	DCDA	12.6 ± 0.4	9.16 ± 0.26	63.2	37.6

a Reported values are averages and standard deviations of analytical replicates.

b Abreviations: cellulose-to-glucose conversion during enzymatic hydrolysis or DCDA (CGC), hemicellulose-to-xylose conversion during enzymatic hydrolysis or DCDA (HXC).

The DCDA thermochemical process produced the highest concentrations of both glucose and xylose among the DM hydrolysates (Table 2). In this process, the cellulose-to-glucose (CGC) and hemicellulose-to-xylose (HXC) conversions were 63.2 and 37.6%, respectively (Table 2), well above other treatments; the HXC was higher than all others except for SPORL (Table 2). However, when SPORL’s yield (80%) is considered, the effective HXC (yield multiplied by HXC) decreases to 36%, making it lower than that of the DCDA method.

Given the higher concentrations of glucose and xylose in the DCDA hydrolysate compared to the other hydrolysates (Table 2), we selected DCDA as the pretreatment method to evaluate microbial fermentations. A characterization of the DCDA hydrolysate (Table 3) shows that the fermentable (soluble) carbohydrates constituted about 43% of the COD in the hydrolysate (Table 3). Acetic acid production during pretreatment was likely the result of hydrolysis of hemicellulose acetyl groups. The measurement of inorganic nutrients indicates the presence of essential micronutrients such as nitrogen, phosphorus, and sulfate. However, the estimated N/C ratio (Table 3) indicates a potential nitrogen deficiency for microbial growth (Rughoonundun et al., 2012).

**TABLE 3:** Average characteristics of DCDA hydrolysate.

Characteristic		Concentration^c^	Fraction of COD	N^d^
Organics (gCOD/L)	COD	71.7 ± 3.2		3
	Soluble COD	55.3 ± 7.8	77.2%	3
	Uncharacterized COD^a^	9.34 ± 3.9	15.7%	2
	Total soluble carbohydrates^a^	42.6 ± 4.0	59.4%	3
	Glucose	12.6 ± 0.4	23.0%	3
	Xylose	9.16 ± 0.26	17.6%	3
	Cellobiose	1.18 ± 0.20	12.8%	2
	Other soluble carbohydrates^a^	23.3 ± 1.62	39.3%	2
	Acetic acid	4.61 ± 0.11	7.78%	2
	Soluble protein	≤0.05	∼0.00%	2
Inorganics (g/L)	Ammonia-N	0.405 ± 0.126		2
	Phosphate-P	0.950 ± 0.124		2
	Sulfate	42.6 ± 8.1		2
	Sodium	23.8 ± 2.3		2
Physical parameters (g/L)	TSS^b^	13.2 ± 1.7		2
	VSS^b^	4.73 ± 0.49		2
	ISS^b^	8.48 ± 1.18		2
	Density	1,060 ± 9		3
N/C^b^ ratio^a^		∼0.018		2

a Calculations: Uncharacterized COD was calculated by subtracting the COD of total soluble carbohydrates and acetic acid from the total measured COD. The COD of soluble carbohydrates was assumed to be 1.122 g COD per g of soluble carbohydrate, which is consistent with a generic disaccharide. Other soluble carbohydrates were determined by substracting the values of glucose, xylose, and cellobiose from total measured soluble carbohydrates. ISS was determined by subtracting VSS from TSS values. To estimate the C value used in the N/C calculation, the decrystallization-dilute acid (DCDA) hydrolysate was assumed to have a COD of 1.067 g COD per g organic material and a molecular fraction of C equal to 0.4, both of which are values consistent with a generic monosaccharide. N/C was calculated to be ammonia concentration (NH4-N) divided by C.

b Abbreviations: total suspended solids (TSS), volatile suspended solids (VSS), inorganic suspended solids (ISS), nitrogen-to-carbon ratio (N/C).

c Data from individual batches are reported in Supplementary Table S3.

d N indicates number of batches used in calculation of reported averages and standard deviations.

### Fermentations of DCDA Hydrolysate

**FIGURE 3:**
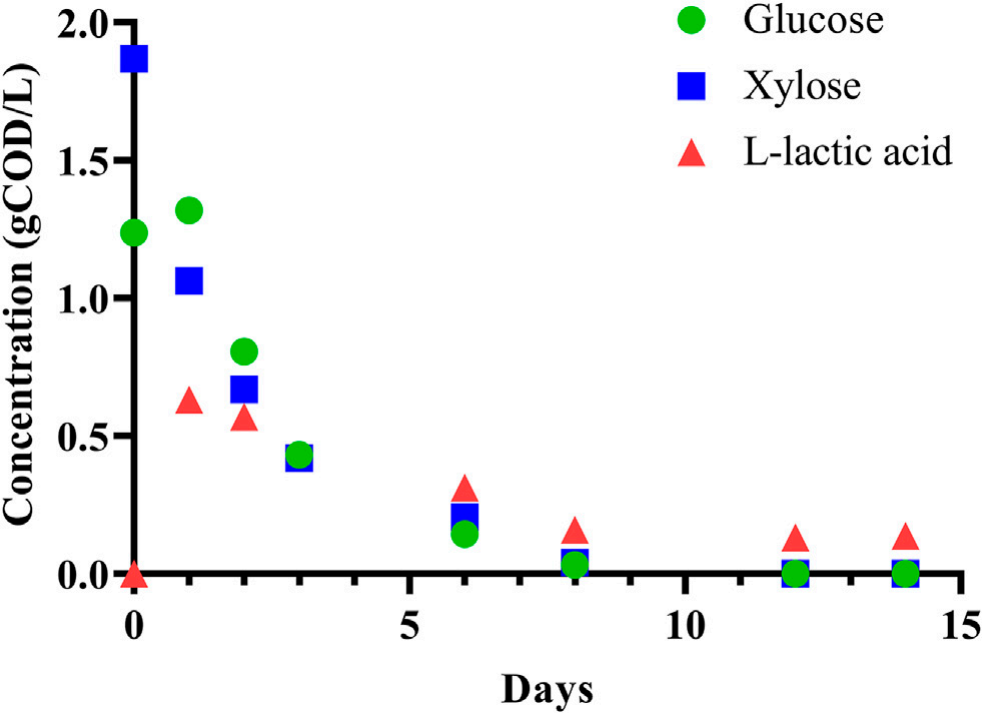
Batch fermentation of DCDA hydrolysate shows simultaneous glucose and xylose consumption with transient accumulation of L-lactic acid.

**FIGURE 4:**
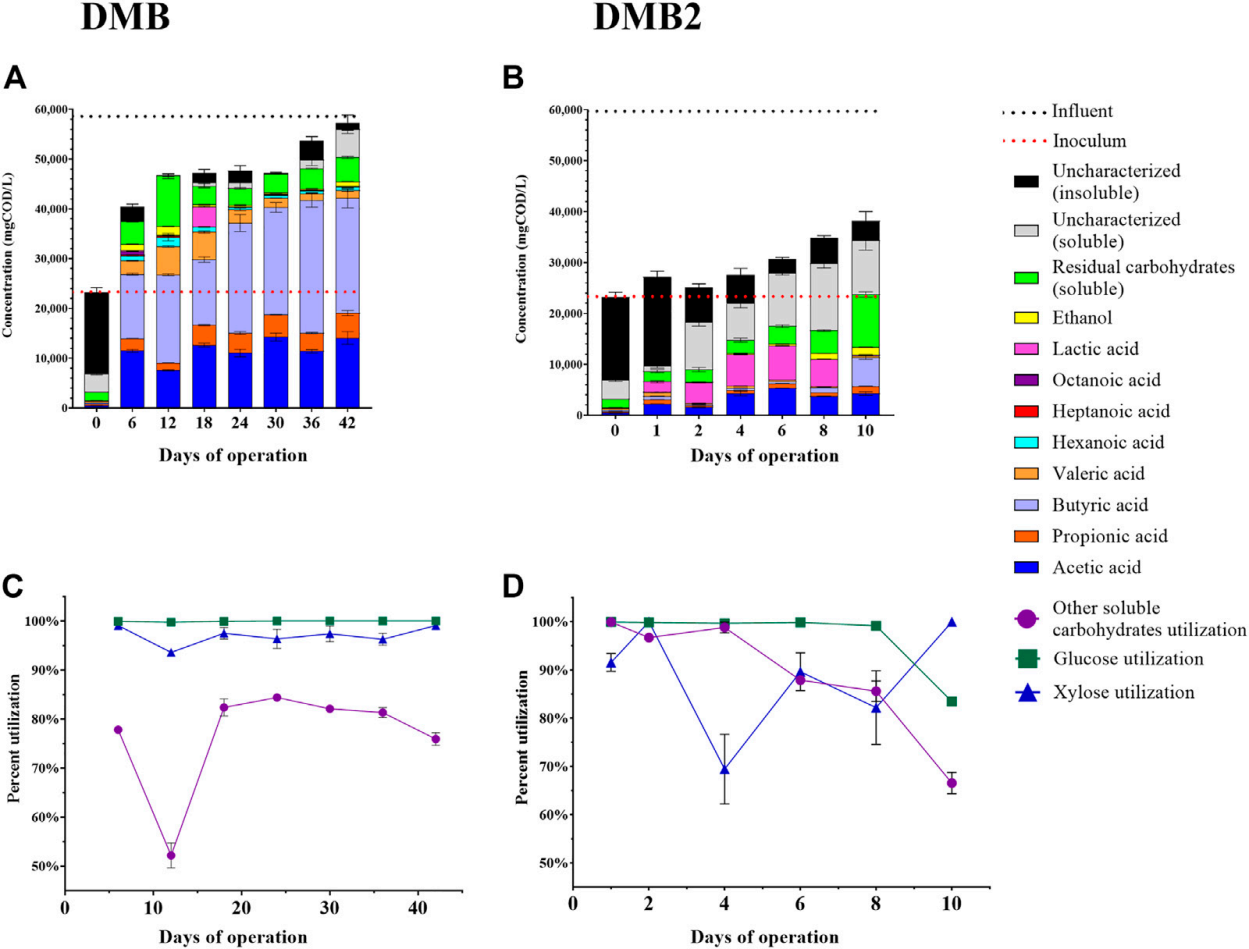
Fermentation experiments with DCDA hydrolysate. One bioreactor experiment (DMB) was ran for 42 days with sampling every 6 days–panels **(A)** and **(C)**. A second bioreactor experiment (DMB2) was ran for 10 days with frequent sampling–panels **(B)** and **(D)**. The concentrations of quantified extracellular metabolites are presented in panels **(A)** and **(B)** for DMB and DMB2, respectively. Bars on Day 0 of panels **(A)** and **(B)** are the measured concentrations at start up and reflect the concentrations in the sludge inoculum. Dashed lines reflect the strength of the influent (black) and inoculum (red) in terms of their COD. The fraction of initial glucose, xylose, and other soluble carbohydrates that was utilized in DMB and DMB2 are presented in panels **(C)** and **(D)**, respectively. Residual soluble carbohydrates accounts for the total concentration of unconsumed soluble carbohydrates including glucose and xylose. Concentrations of other soluble carbohydrates were calculated by subtracting concentrations of glucose and xylose from total soluble carbohydrates. Soluble uncharacterized COD was calculated as the soluble COD subtracted by the combined COD of all measured metabolites. Insoluble uncharacterized COD was calculated as the total uncharacterized COD subtracted by soluble uncharacterized COD. Error bars in all plots reflect the standard deviation of analytical replicates.

To initially assess the fermentability of DCDA hydrolysate, we performed a batch experiment with an inoculum (sludge) to substrate ratio of 7:1 (volume basis) using DCDA hydrolysate (Batch 0 in Supplementary Table S3) as the substrate. We observed complete utilization of glucose and xylose, and transient accumulation of L-lactic acid (Figure 3). Given the evidence of DCDA hydrolysate fermentability, we set up and operated DCDA hydrolysate-fed flow-through bioreactors to test the hypothesis that DM fibers could be used as feedstock for MCFA production. We measured the concentrations of linear short-chain volatile fatty acids (SCFAs) with chain lengths of up to 4 carbons, MCFAs (chain lengths of 5–8 carbons), lactic acid, and ethanol in the inoculum and in the effluent of the bioreactors throughout their operation (Figure 4). Gas production was not measured because in previous fermentation studies with the same sludge inoculum (acid-phase digester) and similar operational conditions we did not detect methanogenic activity nor abundant hydrogen gas production (Scarborough et al., 2018; Fortney et al., 2021).

We operated a first DM bioreactor (DMB) for a total of 42 days, which was fed Batch 1 of DCDA hydrolysate (Supplementary Table S3). At Day 0, the reactor was filled with the sludge inoculum and began receiving the DCDA hydrolysate. The COD in the reactor at start up was ∼23,000 mg/L, the majority of which was insoluble (Figure 4A) and corresponded to the high concentration of cell biomass in the inoculum. Throughout operation the bioreactor received DCDA hydrolysate that had a concentration of ∼58,000 mgCOD/L, and thus, the total COD concentration in the bioreactor increased throughout its operation (Figure 4A). Butyric (C4) and acetic (C2) acids consistently made up the majority of soluble fermentation products (Figure 4A), with C2 concentrations ranging from ∼7,600 to 14,000 mgCOD/L (16–30% as COD_C2_/COD_Total_) and C4 concentrations ranging from ∼13,000 to 27,000 mgCOD/L (28–50% as COD_C4_/COD_Total_), respectively. On Day 12, the highest concentration of total MCFAs was observed at 7,900 mgCOD_MCFAs_/L (17% as mgCOD_MCFAs_/COD_Total_) was achieved wherein valeric acid (C5) and caproic acid (C6) concentrations were 5,700 ± 170 and 1,900 ± 760 mgCOD/L, respectively. An octanoic acid (C8) concentration of 700 ± 40 mgCOD/L was reached on Day 6. The lowest yields of both C2 and propionic acid (C3) also occurred on Day 12, which suggests a microbial competition between production of short-chain and medium-chain fatty acids (Liu et al., 2020). Lactic acid was observed to accumulate on Day 18 (Figure 4A). Ethanol was also observed to transiently accumulate, with a maximum observed concentration of 1,700 ± 53 mgCOD/L or about 3.7% (COD_Ethanol_/COD_Total_) of the total COD in the bioreactor. Carbohydrate utilization was high, with both glucose and xylose consumption being greater than 93%, and the utilization of other soluble carbohydrates being greater than 50% throughout bioreactor operation (Figure 4C).

Because the sampling during the first bioreactor operation was initiated after 6 days of operation, we set up a second reactor (DMB2), which was fed Batch 2 of DCDA hydrolysate (Supplementary Table S3), to investigate the transient behavior during the first 10 days of operation (Figure 4B). In this case, we observed a clear accumulation of lactic acid in the culture broth during the first few days of operation, and the apparent consumption of the accumulated lactic acid by the 10th day, when the reactor was stopped. Ethanol was detected at Days 8 and 10, similar to the detection of this fermentation product in Days 6 and 12 of DMB operation. As with the DMB operation (Figure 4A), the total measured COD inside the DMB2 bioreactor (Figure 4B) reflected a COD increase throughout reactor operation due to the gradual transition from sludge inoculum to the accumulation of fermentation products from metabolism of DCDA hydrolysate. Throughout the duration of DMB2, the insoluble COD (sludge biomass) decreased as the total COD increased (Figure 4B). By Day 10, DMB2 was producing C2 and C4 as the two main end products quantified in the fermention broth (Figure 4B) as was the case for DMB starting at Day 6. However, a characteristic of DMB that is not observed during the short operation of DMB2 was the production of MCFAs, which were detected by Day 6 in DMB. Additionally, uncharacterized soluble COD did not constititue a major fraction of COD in DMB fermentation broths whereas this was the case for DMB2 during the first 10 days of operation (Figure 4B). These observations indicate that both reactors exhibited similar fermentation patterns (total COD, C2 production, C4 production by Day 6 in DMB and Day 10 in DMB2), and there was an apparent time lag in DMB2.

### Analysis of Microbial Communities in DCDA Hydrolysate Fed Bioreactors

**FIGURE 5:**
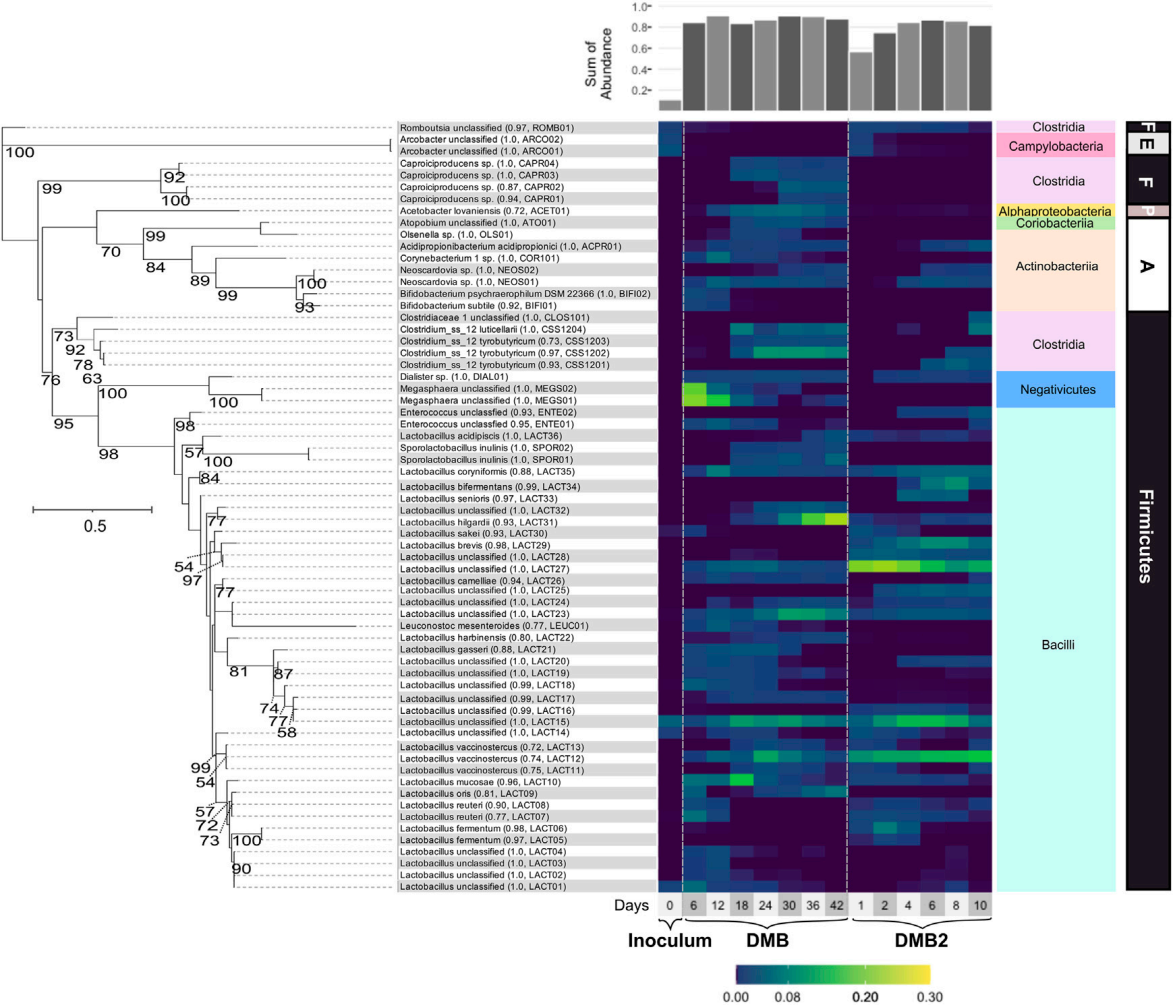
Phylogenetic tree and heatmap of ASVs with relative abundances >1% in the inoculum or at one or more timepoints during DMB and DMB2 operations. Branch labels indicate bootstrap values; values <50 are not shown. The scale bar indicates the branch length (solid-lined) at which 0.5 changes per nucleotide is estimated. Assignments ending in “sp.” indicate an assignment to an uncultured bacterium. Assignments ending in “unclassified” denote ASVs that were unable to be classified at the species-level, and in one case (CLOS101) at the genus-level. Parenthetical numeric values indicate the confidence value of the classification of the species, genus if the species-level is not given, or family if the genus-level is not given. Parenthetical abbreviations are manually assigned monikers. A bar plot of the sum of abundances of displayed assignments is shown atop the heatmap. The phylum (bold) and class (colored boxes) of ASVs are shown right of the heatmap. Abbreviations: Firmicutes (F), Epsilonbacteraeota (E), Proteobacteria (P), Actinobacteria (A).

To determine the composition of the community that is enriched in the DCDA-hydrolysate fed bioreactors, we collected DNA samples for 16S rRNA gene amplicon sequencing. The most abundant ASVs (i.e., relative abundance >1.0% at one or more time points) were affiliated with the phyla Firmicutes, Actinobacteria, Proteobacteria, and Epsilonbacteraeota throughout operation of both bioreactors (Figure 5). The abundance of abundant ASVs in DMB and DMB2 (Figure 5) and ASVs collapsed at the species-level can be seen in Supplementary Tables S4, S5, respectively. Both bioreactors were seeded with sludge inoculum (i.e., 100% of the working volume was inoculum at start up). Abundant ASVs in DMB and DMB2 only accounted for ∼11% of the sludge inoculum’s microbial composition (Supplementary Table S4), which possessed an overall diverse microbial composition (Supplementary Table S5). This diversity can be expected given that acid-phase digesters receive complex, undefined organic material composed of solids deriving from the biological treatment of municipal wastewater. Contrarily, in both bioreactors, which received carbohydrate-rich DCDA hydrolysate, the majority of abundant ASVs were assigned to taxa of lactic acid bacteria. In DMB2, other abundant ASVs represented SCFA producing bacteria, and in DMB, SCFA and MCFA producing bacteria. Generalized least square (GLS) analysis showed correlations among genera, including lactic acid producing bacteria and lactic acid utilizing bacteria, as well as correlations between genera and metabolomic data (Supplementary Table S6).

The most abundant bacteria in both bioreactors were lactic acid bacteria including members of the class Bacilli, within Firmicutes, and of classes Actinobacteria and Coriobacteriia within the Actinobacteria phylum, explaining the transient accumulation of lactic acid during stages of DMB and DMB2 operations. The 16S rRNA-based analysis separated abundant members of *Lactobacillus*, a Bacilli genus, into thirty-six ASVs, many of which were abundant in DMB2 even by Day 1 (Figure 5). There were six *Lactobacillus* ASVs with assigments corresponding among six species described as or closely related to homofermenters, with some being described as faculatively heterofermenative or additionally capable of lactic acid utilization: *L*. *coryniformis*, *L*. *acidipiscis*, *L*. *camelliae, L. gasseri, L. sakei, L. bifermentans* (Kandler et al., 1983; Tanasupawat et al., 2000; Tanasupawat et al., 2007; Bintsis, 2018; Montanari et al., 2018; Abedi and Hashemi, 2020). There were twelve ASVs with assigments corresponding among eight *Lactobacillus* species described as or closely related to heterofermenters: *L*. *vaccinostercus*, *L*. *hilgardii*, *L*. *oris, L. reuteri, L. mucosae, L. harbinensis, L. brevis, L. fermentum* (Vaughn et al., 1949; Farrow and Collins, 1988; Agati et al., 1998; Miyamoto et al., 2005; Oki et al., 2012; Sun Z. et al., 2015; Gaenzle, 2015; Valeriano et al., 2019). The remaining, unclassified *Lactobacillus* ASVs clustered among species of both phenotypes (Figure 5). GLS analysis showed that the *Lactobacillus* genus significantly correlated with glucose utilization, xylose utilization, and total carbohydrate utilization in both reactors, and that this was the only genus significantly correlated with these utilization variables (Supplementary Table S6).

While lactic acid bacteria in DMB2, the short-term reactor, were almost exclusively *Lactobacillus,* DMB additionally enriched for ASVs belonging to other lactic acid producing bacteria in the Bacilli class (Figure 5). Among these ASVs were assignments to *Leuconocostoc mesenteroides* (LEUC01), which is a heterofermentative species (Dols et al., 1997), two ASVs assigned to *Enterococcus* (ENTE01 and ENTE02), whose cultured representatives are described as homofermenative (Abdel-Rahman et al., 2011; Subramanian et al., 2015), and two ASVs assigned as *Sporolactobacillus inulinis* (SPOR01 and SPOR02). Cultured representatives of *S. inulinis* show that it is a sporeforming, heat-resistant, homofermentative lactic acid bacteria (Kitahara and Lai, 1967). LEUC01 most closely clustered with presumably heterofermentative *Lactobacillus* ASVs, and ENTE01, ENTE02, SPOR01, and SPOR02 most closely clustered with presumably homofermentative *Lactobacillus* ASVs (Figure 5). Another group characteristically described as lactic acid bacteria present in DMB were members of the phylum Actinobacteria including ASVs assigned to genera *Bifidobacterium*, *Neoscardovia*, *Olsenella* (Dewhirst et al., 2001), and *Atopobium* (Jovita et al., 1999) (Figure 5). *Bifidobacterium* and *Neoscardovia* are genera within the family *Bifidobacteriaceae*, which are notable for the possession of the “bifid shunt”, a pathway involving the fermentation of carbohydrates into lactic acid and acetic acid (Levantovsky et al., 2018)*.*

Several ASVs present in the microbial community in high abundance are known lactic acid utilizing bacteria. For instance, in DMB two abundant ASVs (MEGS01 and MEGS02) were assigned to *Megasphaera* (Figure 5)*,* a Firmicute in the Negativicutes class that has been described as consuming lactic acid and performing odd- and even-chain elongating metabolism that leads to MCFA production (Marounek et al., 1989; Weimer and Moen, 2013; Kim et al., 2019). However, *Megasphaera* were not enriched during the operation of DMB2. According to GLS analysis, the presence of the *Megasphaera* genus was significantly correlated with C7 and C8 production in the DMB, the first bioreactor (Supplementary Table S6). Another significant correlation existed between the *Bifidobacterium* and *Megasphaera* (Supplementary Table S6). After *Megasphaera*’s abundance in the DMB decreased, other lactic acid utilizing bacteria increased in abundance, such as the four ASVs assigned to *Caproiciproducens* (Figure 5), a Firmicute in the Clostridia class (Contreras-Dávila et al., 2020; Esquivel-Elizondo et al., 2021). *Caproiciproducens* has been shown to produce intracellular lactic acid and then use it to produce C4 and MCFAs when sugars are the initial substrate (Esquivel-Elizondo et al., 2021) and is also proposed to be able to utilize lactic acid as an initial substrate (Contreras-Dávila et al., 2020; Gao et al., 2021). In both reactors, ASVs assigned to another Clostridia genus, *Clostridium sensu stricto 12*, began to accumulate after several days of reactor operation (Figure 5). Given that *Clostridium butyricum*, a butyrate fermenter, serves as type species of *Clostridium sensu stricto* (Lawson and Rainey, 2016), ASVs assigned to *Clostridium sensu stricto 12* are presumed to likewise produce C4. GLS analysis of DMB, showed both *Caproiciproducens* and *Clostridium sensu stricto 12* being signficantly correlated with C4 (Supplementary Table S6). Other statistically significant correlations are found between *Caproiciproducens* and *Clostridium sensu stricto 12* with *Sporolactobacillus*. *Caproiciproducens* and *Clostridium sensu stricto 12* are also significantly correlated with *Acetobacter* (Supplementary Table S6), a genus characterized as an acetic acid producer (Cleenwerck et al., 2002). *Acidipropionibacterium,* an apparent lactic utilizing bacteria (Luo et al., 2017; Candry et al., 2020), correlated with *Lactobacillus*.

**FIGURE 6:**
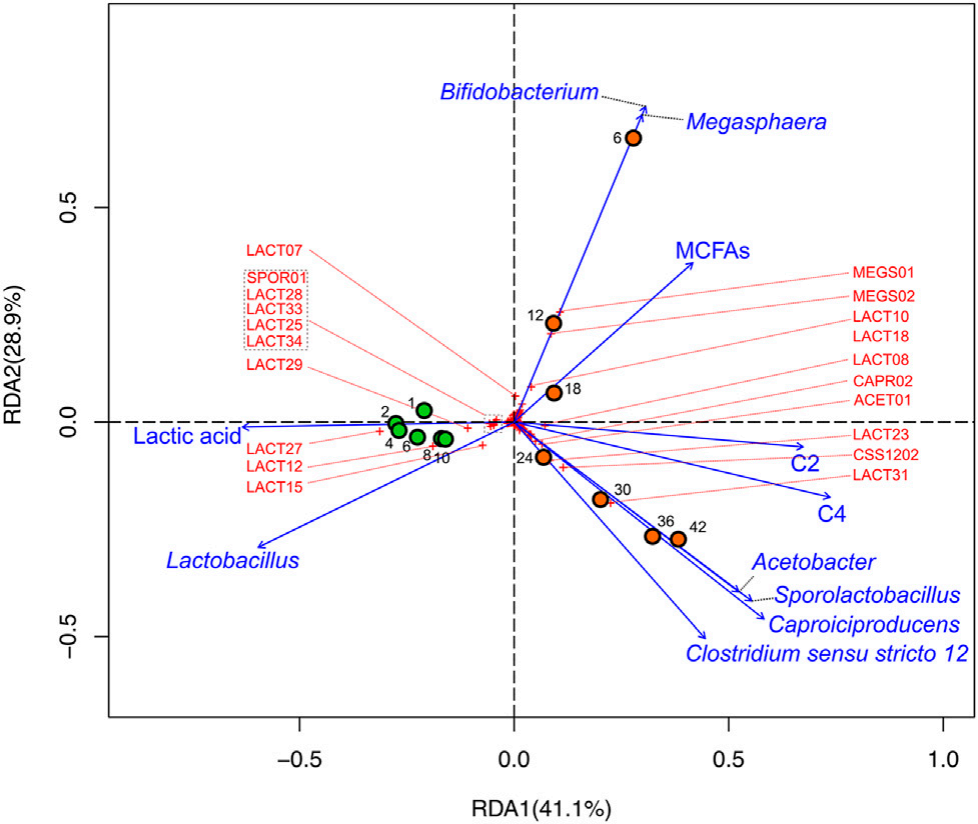
RDA biplot illustrating microbial and metabolomic data of DMB and DMB2. Circles represent sample points that were taken during reactor operation (orange: DMB; green: DMB2), numbers adjacent to circles indicate the sampling day to which the circles correspond, crosses to ASVs (ASVs that are ordinated away from the center are labeled by their monikers in red; Supplementary Table S4), and vectors to explanatory variables. The percentage of total variation that each axis represents is indicated in parentheses within the axis titles.

To further evaluate the relations between community members and fermentation products, we employed redundancy analysis (RDA) as a tool to explore the variation of response variables (i.e., relative abundances of all ASVs) with explanatory variables by multivariate multiple linear regressions (Legendre and Gallagher, 2001). As explanatory variables we used metabolomic yields of MCFA, C2, C4, lactic acid and relative abundances of *Lactobacillus*, *Sporolactobacillus*, *Bifidobacterium*, *Megasphaera*, *Caproiciproducens*, *Clostridium sensu stricto 12*, and *Acetobacter* (Figure 6).

The analysis showed that all timepoints of DMB2 were associated with *Lactobacillus* and lactic acid (Figure 6), which we would expect given accumulation of lactic acid (Figure 4B) and the abundance of *Lactobacillus* ASVs in the reactor (Figure 5). Timepoints from DMB2 do not possess any meaningful association with *Megasphaera* nor with MCFAs nor do they trend toward such (Figure 6). In contrast, the analysis suggests a strong correlation of *Megasphaera* and *Bifidobaterium* with MCFAs in the early timepoints of DMB operation as can be seen in the top-right quadrant of Figure 6. The RDA also show that during the later part of DMB operation, *Caproiciproducens, Clostridium sensu stricto 12*, *Sporolactobacillus*, and *Acetobacter* were closely ordinated and correlated with the explanatory variables C2 and C4 (Days 24, 30, 36, and 42 in Figure 6), The correlations between lactic acid producing *Sporolactobacillus* and lactic acid utilizing genera *Caproiciproducens* and *Clostridium senso stricto 12* agrees with observations from the GLS correlation analysis mentioned above (Supplementary Table S6).

## Discussion

Typically, the utilization of bioreactors by dairy operations is limited to methanogenic anaerobic digesters, but here we have presented data from bioreactors that produced SCFAs and MCFAs (Figure 4) from pretreated DM fibers, and likely used lactic acid as an intermediate metabolite. As a lignocellulosic biomass, a DM-to-MCFA bioprocessing scheme must include the following steps (Agler et al., 2011): the breakdown of DM polysaccharides into readily fermentable carbohydrates (i.e., sugars), primary fermentation, (i.e., the fermentation of carbohydrates into products that are able to be further reduced), and the chain elongation of primary fermentation products such as lactic acid - alternative, chain elongation can occur directly through sugar metabolism. The first step was evaluated by applying chemical-enzymatic and thermochemical unit operations onto physically treated DM and the latter two were carried out by an acidogenic mesophilic mixed community. These steps are not conventional to dairy operations, but may be of economic and environmental interest.

Given the ongoing research on the roles of pretreatment strategies on the breakdown of lignocellulosic biomass, we elected to explore dilute acid- (DA and SPORL), sulfonation- (SPORL), and alkaline-based (Alkaline, Cu-AHP, and CAP) chemical pretreatments prior to enzymatic hydrolysis. Weak acids can increase the exposure of cellulose in lignocellulosic biomass primarily through lignin penetration, pre-hydrolysation of cellulose, and hemicellulose solubilization (Lee et al., 1999; Zaldivar et al., 2001), all of which increase the surface area of cellulose upon which cellulasic activity can occur. Indeed, we observed improved CGCs during enzymatic hydrolysis of DA and SPORL pretreated DM fibers when compared to untreated DM fibers (Table 2). Additionally, both DA and SPORL led to increases in HXCs, indicating that the enzymatic hydrolysis of hemicellulose in these pretreated samples can benefit from DA and SPORL pretreatments. SPORL pretreatment can decrease lignin hydrophobicity through lignin sulfonation, which reduces the ability of lignin to act as an enzyme adsorbent (Pan et al., 2005; Shuai et al., 2010; Yang et al., 2015). This additional chemistry may be the reason SPORL led to the highest CGC and HXC when compared to all other treatment types in Scheme 1 (Table 2). Furthermore, these observations support other studies where SPORL was found to be a superior chemical pretreatment when applied to DM (Kim and Karthikeyan, 2021). Alkaline-based pretreatments can solubilize hemicellulose and lignin (Kim et al., 2016). Lignin can also be oxidized with the use of an oxidant such as hydrogen peroxide (Su et al., 2015). Furthermore, the supplement of metallic catalysts have been found modify lignin in a variety of biomass dependent manners (Li et al., 2013; Bhalla et al., 2016), and thereby increase enzymatic hydrolysability. We selected the pretreatment conditions (i.e., chemical loadings, ambient temperature, and duration) of Alkaline, Cu-AHP, and CAP pretreatments on a previous study conducted on hybrid poplar (Bhalla et al., 2016). Under these conditions, the solubilization of hemicellulose in DM fibers was apparent during all alkaline-based chemical pretreatment (Table 1) and statistically significant (Supplementary Table S1); however, the solubilization of lignin was not. The enzymatic hydrolysis of DM fibers using these three chemical pretreatments showed and initial increase in glucose and xylose concentrations (Supplementary Table S2), but these sugars were not stable under the pretreatment conditions (Supplementary Table S2) making these alkaline processes not suitable for downstream fermentation of DM fiber hydrolysates.

The DCDA pretreatment (Scheme 2) was found to be advtangeous over chemical-enzymatic pretreatments (Scheme 1) due to its higher biomass conversion rates, which we interpret as the efficiency of DM fiber breakdown, and resultant hydrolysate concentrations of glucose and xylose (Table 2), which should be maximized as they serve as substrates for downstream fermentation. Liao et al. (2006) reported a higher CGC (∼90%) from raw DM fibers with DCDA than this study did, suggesting that DCDA methods in this study could be improved. Particularly, the temperature in Liao et al. during the dilute acid step was higher (135°C compared to 103°C) and may have been the differentiating parameter, especially since increases in temperature at the same acid concentration used in this study during the dilute acid step (12.5%) were concluded to lead to higher concentrations of sugars during the thermochemical treatement of DM (Liao et al., 2006). We were unable to achieve a temperature of 135°C with the vessel (boiler flask) used in our methods during this process and speculate Liao et al. used a pressurized vessel; however, this was unspecified (Liao et al., 2006) – the HXC was not reported.

Conceptually, DM handling facilities could integrate Scheme 2 alongside anaerobic digesters (ADs). Raw DM could first undergo separation, with the liquid fraction directed to an AD and the solid fraction directed to Scheme 2. Alternatively, Scheme 2 could be applied post-AD, receiving digester effluent. In both scenarios, the solid fraction that is directed out of Scheme 2 (unit operation #5 in Figure 1) could be redirected to AD to be used for further biogas production. Considering the post-AD scenario, future experiments could involve the mixed fermentation of hydrolysates produced when Scheme 2 is applied to fibers of anaerobically digested DM. Anaerobically digested DM biomass is more cellulosic than raw DM (Yue et al., 2010). Such a difference would lead to a DCDA hydrolsate that differs from this study’s, which could possibly impact the hypothetical downstream fermentation’s end product profile. Further evaluation of these abiotic steps requires analyses beyond the scope of this study. However, in the below section we discuss the metabolic features of DCDA hydrolysate fermentation, imperative to the DM-to-MCFA pathway and of relevance to the fermentation of carbohydrate-rich feedstocks.

### Primary Fermentation Products Lactic Acid and C2 are Chain Elongated to MCFAs and C4 During DCDA Hydrolysate Metabolism

Results from all bioreactors indicate that DCDA hydrolysate can support activity of a saccharolytic, lactic acid producing, and hexose, pentose, and lactic acid utilizing microbial community under mesophilic conditions. Despite the evident enrichment of lactic acid bacteria in DMB and DMB2 (Figure 5), lactic acid accumulation was relatively low in the flow-through bioreactors (Figures 4A,B). Thus, in conjunction with presence of lactic acid utilizing bacteria (Figure 5) and GLS and redundancy analysis, we hypothesize that lactic acid was a key intermediate in DCDA hydrolsate fermentation, particularly during MCFA and C4 production.

Most notable of the lactic acid bacteria was the genus *Lactobacillus,* which significantly correlated with total carbohydrate utilization rates (Supplementary Table S6), dominated abundance in both DMB and DMB2 (Figure 5), and whose ASVs were closely associated with all time points and explanatory variables (Figure 6). These observations indicate that *Lactobacillus* play major a role in the carbohydrate degradation of DCDA hydrolysates, and are in agreement with *Lactobacillus* being associated with non-methanogenic mixed microbial fermentations when substrates are rich in carbohydrates such as lactose (Xu et al., 2018), pentoses (Andersen et al., 2017; Scarborough et al., 2018), and readily degradable polysaccharides (Andersen et al., 2015; Contreras-Dávila et al., 2020; Liu et al., 2020; Gao et al., 2021). The moderate C2 yields measured at all DMB timepoints (Figure 4A) is supported by the constant presence of heterolactic fermenters such as *Lactobacillus*, *Leuconocostoc*, *Atopobium*, *Olsenella*, and *Bifidobacteriaceae* as well as the acetogenic *Acetobacter*. As with *Lactobacillus*, the C2 producing lactic acid bacteria *Bifidobacterium*, *Atopobium*, and *Olsenella* have been reported as abundandant members among various acidogenic communities fed carbohydrate-rich feedstocks (Scarborough et al., 2018; Lambrecht et al., 2019; Liu et al., 2020). Given the evident significance of lactic acid bacteria during DCDA hydrolysate fermentation, we hypothesize that the abundant lactic acid utilizing bacteria such as *Megasphaera, Caproiciproducens*, and *Clostridium sensu stricto 12* consume the intermediate lactic acid.

The only exception to *Lactobacillus* as the most abundant genus of DMB occurred on Day 6 when MEGS01 and MEGS02 represented 21 and 20% relative abundance, respectively (Supplementary Table S4). The high abundance of *Megasphaera* in early DMB operation and decline thereafter may explain the higher accumulation of MCFAs at the beginning of DMB operation (Figure 4A) given the ability of lactic acid based chain elongation in some of its members and the genus’s correlations in GLS analysis (Supplementary Table S6). This notion is further supported by findings in RDA where *Megasphaera*, *Bifidobacterium*, and MCFAs were in close association with one another (Figure 6) indicating *Megasphaera* may have used lactic acid produced by *Bifidobacterium* for chain elongation. Thus, MEGS01 and MEGS02 were likely responsible for MCFA production in DMB. The critical role of lactic acid utilizing *Megasphaera* in MCFA production during mixed fermentations of carbohydrate-rich feedstocks is a metabolic feature this study shares with other studies where a carbohydrate fermenting bioreactor operated at a slightly acidic pH and mesophilic temperature (Andersen et al., 2015; Scarborough et al., 2018). In both referenced studies, as in this one, the early enrichment and subsequent wash-out of *Megasphaera* was observed, indicating that *Megasphaera* spp. may be outcompeted under these simple conditions in single-system mixed fermentations of carbohydrate-rich feedstocks. It should be noted that in Andersen et al., transient *Megasphaera* enrichment was able to be achieved with the aid of exogenous hydrogen generated from membrane electrolysis (Andersen et al., 2015), which was hypothesized to be utilized to produce MCFAs. *Megasphaera* were notably absent from DMB2 (Figure 5) despite being operationally similar to and seeded with the same sludge inoculum as DMB (Figure 5). Additionally, DMB2 did not demonstrate any significant MCFA production (Figure 4). From these observations, we offer the thought that sustained MCFA production during fermentations of DM fiber hydrolysates might coincide with sustained presence of *Megasphaera* that emerge as early abundant microbial community members, and that the sustained enrichment of *Megasphaera* requires additional considerations to the design of the bioreactor system.

The main end product throughout DMB operation was C4 (Figure 4A) as is the case in several afore referenced experiments involving carbohydrate fed bioreactors whose operating conditions are similar to ours. ASVs within the two abundant Clostridia genera, *Clostridium sensu stricto 12* and *Caproiciproducens*, were likely responsible for C4 production in later days of DMB operation and Day 10 of DMB2 given that they were the only members capable of such. In agreement with this inference, the two genera have recently been associated with SCFA production from xylose as a sole carbon source and electron donor in a mesophilic mixed community at a pH of 5.5, as in DMB (Qian et al., 2020). Additionally, Gao et al. showed that *Caproiciproducens* may utilize lactic acid, C2, and ethanol for the production of C4 and C6 (Gao et al., 2021). This is further supported by results from our statistical analyses, which in addition to C4 showed a close association of the two genera with *Sporolactobacillus* and *Acetobacter* (bottom-right quadrant of Figure 6). The association of *Clostridium sensu stricto 12* and *Caproiciproducens* with *Sporolactobacillus* suggests another example of a lactic acid production and utilization between two groups of organisms, which in this case was for the production of C4. The association of *Clostridium sensu stricto 12* and *Caproiciproducens* with *Acetobacter* in this study might suggest a role of C2 in the microbial networks that formed during DCDA hydrolysate fermentation. Scarborough et al. used a metabolic model to explore the potential effect of C2 co-utilization during chain elongation (Scarborough et al., 2020). The metabolic model predicted C4 to be the sole product during utilization of acetic acid and lactic acid at high acetic acid:lactic acid ratios (greater than 0.96, as is the case in this study). The same model predicts no energetic benefit in co-utilizing C2 with lactic acid as opposed to lactic acid being the sole substrate. However, Liu et al. proposed *Clostridium sensu stricto 12* to be able to perform such a co-utilization (Liu et al., 2020). Furthermore, an experiment where organic substrate exclusively consisted of C2 and lactic acid was shown to enrich for both *Clostridium sensu stricto 12* and *Caproiciproducens* while producing C4 (Detman et al., 2021). Thus, the co-utilization of C2 and lactic acid for the production of C4 may indeed have occurred in the DMB bioreactor.

The economic viability of the DM-to-MCFA pathway would increase with increases in MCFA selectivity and productivity during DCDA hydrolysate fermentation, which mostly produced C2 and C4 rather than MCFAs. Such improvement would involve engineering strategies to select for specific organisms. We referenced the strategy of membrane electrolysis, which selected for *Megasphaera* and improved MCFA production as a result of abiotically generated hydrogen (Andersen et al., 2015). Another strategy is the compartmentalization of lactic acid producing bacteria and lactic acid utilizing bacteria, which was recently shown to improve MCFA production from acid whey waste due to the high selectivity of lactic acid in the first compartment (Xu et al., 2018). Yet another strategy might be the curation of a synthetic microbiome in lieu of allowing fermentative mixed communities to self-assemble. For instance, given the isolation techniques that have been described for homofermentative *S. inulinus* (Yu et al., 2011) and for MCFA producing *Caproiciproducens* spp. (Esquivel-Elizondo et al., 2021), a synthetic microbiome of isolate representatives of these bacteria could theoretically recover all carbohydrate derived electron equivalents as intermediate lactic acid, which could then be chain-elongated to C6, producing CO_2_ as a byproduct (based solely on the proposed metabolic pathways involved in these organisms).

## Concluding Remarks

In this study, we demonstrated a novel bioprocessing pathway in which DM was converted into SCFA and MCFA. A comparison of pretreatment processes for DM indicated that the thermochemical DCDA process yielded the highest combined glucose and xylose concentrations and did not require enzymatic treatment to produce carbohydrate-rich hydrolysates. Mixed fermentations of DCDA hydrolysate involved the near complete utilization of glucose and xylose, with lactic acid emerging as the key intermediate in the transformation of sugars to SCFA and MCFA. Statistical analyses such as GLS and RDA supported a hypothesis that the microbial community network was based on the co-existence of abundant lactic acid producing bacteria and lactic acid utilizing bacteria. In early stages of reactor operation, this co-existence lead to accumulation of MCFA with *Bifidobacterium* and *Lactobacillus* as the main lactic acid producing organisms and *Megasphaera* as the main lactic acid utilizer and MCFA producer. Later in reactor operation, C4 and C2 were major end products of DCDA hydrolysate metabolism. The statistical analyses supported the hypothesis that under the later operation conditions *Lactobacillus* and *Sporolactobacillus* were the main lactic acid producing bacteria and that *Clostridium senso stricto 12* and *Caproiciproducens* were the primary C4 producers, who may have co-utilized lactic acid and C2. After the demonstration that MCFA can be produced from DM fibers presented here, future studies could be directed to identifying areas for optimization and analyzing the economic viability of using DM fibers for MCFA production. Additionally, a more in-depth understanding of the metabolism of the identified microbial community members may aid in further refining their role in MCFA production from DCDA hydrolysates.

## Data Availability

The datasets presented in this study can be found in online repositories. The names of the repository/repositories and accession number(s) can be found below: https://www.ncbi.nlm.nih.gov/sra/PRJNA721738.
